# Supplementary material for: Evaluation of quantitative biosensor for glucose-6-phosphate dehydrogenase activity detection
Source: PLoS One. 2019 Dec 20;14(12):e0226927. doi: 10.1371/journal.pone.0226927 (PMC6924682; doi:10.1371/journal.pone.0226927)
Supplement: S1 Table — Results of RBC count, Hb and Hct in males were significantly higher than females. (DOCX) [file pone.0226927.s001.docx]

S1 Table.

| **CBC parameters** | **Total**  **(n=216)** | **Male**  **(n=130)** | **Female**  **(n=86)** | ***p*-value** |
| --- | --- | --- | --- | --- |
| RBC count (x10^6^/µL) | 4.93 ± 0.75 | 4.81 ± 0.73 | 5.11± 0.75 | **0.003** |
| Hemoglobin (Hb; g/dL) | 16.4 ± 2.58 | 15.9 ± 2.58 | 17.2 ± 2.39 | **0.000** |
| Hematocrit (Hct; %) | 49.3 ± 7.74 | 47.7 ± 7.64 | 51.6 ± 7.35 | **0.000** |
| Mean corpuscular volume (MCV; fL) | 100.1 ± 8.01 | 99.5 ± 8.99 | 101.1 ± 6.16 | 0.165 |
| Mean corpuscular hemoglobin (MCH; pg) | 33.4 ± 2.98 | 33.1 ± 3.33 | 33.8 ± 2.32 | 0.126 |
| Mean corpuscular hemoglobin concentration (MCHC; %) | 33.3 ± 0.85 | 33.3 ± 0.94 | 33.4 ± 0.68 | 0.462 |

*. The significant (Sig.) < 0.05 level determined a significantly difference.
