# Supplementary material for: Evaluation of quantitative biosensor for glucose-6-phosphate dehydrogenase activity detection
Source: PLoS One. 2019 Dec 20;14(12):e0226927. doi: 10.1371/journal.pone.0226927 (PMC6924682; doi:10.1371/journal.pone.0226927)
Supplement: S2 Table — The G-6-PD enzyme activity at certain Hct was subtracted with another getting the different of G-6-PD activity. The mean difference of G-6-PD activity measures the absolute difference between the mean values in two groups. (DOCX) [file pone.0226927.s002.docx]

S2 Table.

| **Hct** | **Compared Hct** | **Mean Difference (%)** | **Standard Error** | **Sig.** |
| --- | --- | --- | --- | --- |
|  |  |  |  |  |
| 30% | 40% | -1.943 | 0.247 | **<0.0001*** |
|  | 50% | -3.470 | 0.287 | **<0.0001*** |
|  | 60% | -4.216 | 0.330 | **<0.0001*** |
| 40% | 30% | 1.943 | 0.247 | **<0.0001*** |
|  | 50% | -1.527 | 0.181 | **<0.0001*** |
|  | 60% | -2.273 | 0.251 | **<0.0001*** |
| 50% | 30% | 3.470 | 0.287 | **<0.0001*** |
|  | 40% | 1.527 | 0.181 | **<0.0001*** |
|  | 60% | -0.746 | 0.229 | **0.0149*** |
| 60% | 30% | 4.216 | 0.330 | **<0.0001*** |
|  | 40% | 2.273 | 0.251 | **<0.0001*** |
|  | 50% | 0.746 | 0.229 | 0.0149 |

*. The significant (Sig.) < 0.05 level determined a significantly difference.
