# Supplementary material for: Evaluation of quantitative biosensor for glucose-6-phosphate dehydrogenase activity detection
Source: PLoS One. 2019 Dec 20;14(12):e0226927. doi: 10.1371/journal.pone.0226927 (PMC6924682; doi:10.1371/journal.pone.0226927)
Supplement: S3 Table — The mean difference measures the absolute difference between the mean value in two groups. (DOCX) [file pone.0226927.s003.docx]

S3 Table.

| **Storage time** | | **Mean Difference** | **Standard Error** | **Sig.** |
| --- | --- | --- | --- | --- |
| **Time (Hr.)** | **Compared Time (Hr.)** |  |  |  |
| **At room temperature** | | | | |
| Immediate | 1 hr | -0.0703 | 0.156 | 1.0000 |
|  | 2 hr | 0.132 | 0.219 | 1.0000 |
|  | 6 hr | -0.0865 | 0.182 | 1.0000 |
|  | 12 hr | 0.295 | 0.183 | 1.0000 |
|  | 24 hr | 0.365 | 0.198 | 1.0000 |
|  | 48 hr | 0.484 | 0.216 | 0.8687 |
|  | 72 hr | 1.143 | 0.237 | **0.0007*** |
| 1 hr | Immediate | 0.0703 | 0.156 | 1.0000 |
|  | 2 hr | 0.203 | 0.232 | 1.0000 |
|  | 6 hr | -0.0162 | 0.157 | 1.0000 |
|  | 12 hr | 0.365 | 0.205 | 1.0000 |
|  | 24 hr | 0.435 | 0.208 | 1.0000 |
|  | 48 hr | 0.554 | 0.247 | 0.8753 |
|  | 72 hr | 1.214 | 0.249 | **0.0006*** |
| 2 hr | Immediate | -0.132 | 0.219 | 1.0000 |
|  | 1 hr | -0.203 | 0.232 | 1.0000 |
|  | 6 hr | -0.219 | 0.206 | 1.0000 |
|  | 12 hr | 0.162 | 0.224 | 1.0000 |
|  | 24 hr | 0.232 | 0.216 | 1.0000 |
|  | 48 hr | 0.351 | 0.221 | 1.0000 |
|  | 72 hr | 1.011 | 0.266 | **0.0149*** |
| 6 hr | Immediate | 0.0865 | 0.182 | 1.0000 |
|  | 1 hr | 0.0162 | 0.157 | 1.0000 |
|  | 2 hr | 0.219 | 0.206 | 1.0000 |
|  | 12 hr | 0.381 | 0.217 | 1.0000 |
|  | 24 hr | 0.451 | 0.189 | 0.6331 |
|  | 48 hr | 0.570 | 0.238 | 0.6140 |
|  | 72 hr | 1.230 | 0.264 | **0.0012*** |
| 12 hr | Immediate | -0.295 | 0.183 | 1.0000 |
|  | 1 hr | -0.365 | 0.205 | 1.0000 |
|  | 2 hr | -0.162 | 0.224 | 1.0000 |
|  | 6 hr | -0.381 | 0.217 | 1.0000 |
|  | 24 hr | 0.0703 | 0.222 | 1.0000 |
|  | 48 hr | 0.189 | 0.215 | 1.0000 |
|  | 72 hr | 0.849 | 0.212 | **0.0083*** |
| 24 hr | Immediate | -0.365 | 0.198 | 1.0000 |
|  | 1 hr | -0.435 | 0.208 | 1.0000 |
|  | 2 hr | -0.232 | 0.216 | 1.0000 |
|  | 6 hr | -0.451 | 0.189 | 0.6331 |
|  | 12 hr | -0.0703 | 0.222 | 1.0000 |
|  | 48 hr | 0.119 | 0.183 | 1.0000 |
|  | 72 hr | 0.778 | 0.265 | 0.1611 |
| 48 hr | Immediate | -0.484 | 0.216 | 0.8687 |
|  | 1 hr | -0.554 | 0.247 | 0.8753 |
|  | 2 hr | -0.351 | 0.221 | 1.0000 |
|  | 6 hr | -0.570 | 0.238 | 0.6140 |
|  | 12 hr | -0.189 | 0.215 | 1.0000 |
|  | 24 hr | -0.119 | 0.183 | 1.0000 |
|  | 72 hr | 0.659 | 0.203 | 0.0692 |
| 72 hr | Immediate | -1.143 | 0.237 | **0.0007*** |
|  | 1 hr | -1.214 | 0.249 | **0.0006*** |
|  | 2 hr | -1.011 | 0.266 | **0.0149*** |
|  | 6 hr | -1.230 | 0.264 | **0.0012*** |
|  | 12 hr | -0.849 | 0.212 | **0.0083*** |
|  | 24 hr | -0.778 | 0.265 | 0.1611 |
|  | 48 hr | -0.659 | 0.203 | 0.0692 |
| **At 4°C** | | | | |
| Immediate | 1 hr | .216 | 3.216 | 1.000 |
|  | 2 hr | 2.351 | 3.216 | 0.996 |
|  | 6 hr | .378 | 3.216 | 1.000 |
|  | 12 hr | 2.432 | 3.216 | .995 |
|  | 24 hr | .351 | 3.216 | 1.000 |
|  | 48 hr | -.351 | 3.216 | 1.000 |
|  | 72 hr | 2.081 | 3.216 | 0.998 |
| 1 hr | Immediate | -.216 | 3.216 | 1.000 |
|  | 2 hr | 2.135 | 3.216 | 0.998 |
|  | 6 hr | .162 | 3.216 | 1.000 |
|  | 12 hr | 2.216 | 3.216 | 0.997 |
|  | 24 hr | .135 | 3.216 | 1.000 |
|  | 48 hr | -.568 | 3.216 | 1.000 |
|  | 72 hr | 1.865 | 3.216 | 0.999 |
| 2 hr | Immediate | -2.351 | 3.216 | 0.996 |
|  | 1 hr | -2.135 | 3.216 | 0.998 |
|  | 6 hr | -1.973 | 3.216 | 0.999 |
|  | 12 hr | .081 | 3.216 | 1.000 |
|  | 24 hr | -2.000 | 3.216 | 0.999 |
|  | 48 hr | -2.703 | 3.216 | 0.991 |
|  | 72 hr | -.270 | 3.216 | 1.000 |
| 6 hr | Immediate | -.378 | 3.216 | 1.000 |
|  | 1 hr | -.162 | 3.216 | 1.000 |
|  | 2 hr | 1.973 | 3.216 | 0.999 |
|  | 12 hr | 2.054 | 3.216 | 0.998 |
|  | 24 hr | -.027 | 3.216 | 1.000 |
|  | 48 hr | -.730 | 3.216 | 1.000 |
|  | 72 hr | 1.703 | 3.216 | 0.999 |
| 12 hr | Immediate | -2.432 | 3.216 | 0.995 |
|  | 1 hr | -2.216 | 3.216 | 0.997 |
|  | 2 hr | -.081 | 3.216 | 1.000 |
|  | 6 hr | -2.054 | 3.216 | 0.998 |
|  | 24 hr | -2.081 | 3.216 | 0.998 |
|  | 48 hr | -2.784 | 3.216 | 0.989 |
|  | 72 hr | -.351 | 3.216 | 1.000 |
| 24 hr | Immediate | -.351 | 3.216 | 1.000 |
|  | 1 hr | -.135 | 3.216 | 1.000 |
|  | 2 hr | 2.000 | 3.216 | 0.999 |
|  | 6 hr | .027 | 3.216 | 1.000 |
|  | 12 hr | 2.081 | 3.216 | 0.998 |
|  | 48 hr | -.703 | 3.216 | 1.000 |
|  | 72 hr | 1.730 | 3.216 | 0.999 |
| 48 hr | Immediate | .351 | 3.216 | 1.000 |
|  | 1 hr | .568 | 3.216 | 1.000 |
|  | 2 hr | 2.703 | 3.216 | 0.991 |
|  | 6 hr | .730 | 3.216 | 1.000 |
|  | 12 hr | 2.784 | 3.216 | 0.989 |
|  | 24 hr | .703 | 3.216 | 1.000 |
|  | 72 hr | 2.432 | 3.216 | 0.995 |
| 72 hr | Immediate | -2.081 | 3.216 | 0.998 |
|  | 1 hr | -1.865 | 3.216 | 0.999 |
|  | 2 hr | .270 | 3.216 | 1.000 |
|  | 6 hr | -1.703 | 3.216 | 0.999 |
|  | 12 hr | .351 | 3.216 | 1.000 |
|  | 24 hr | -1.730 | 3.216 | 0.999 |
|  | 48 hr | -2.432 | 3.216 | 0.995 |

*. The significant (Sig.) < 0.05 level determined a significantly difference.
